# Supplementary material for: Syndromic surveillance during 2022 Uganda Martyrs’ commemoration
Source: PLOS Glob Public Health. 2024 Jan 25;4(1):e0002068. doi: 10.1371/journal.pgph.0002068 (PMC10810525; doi:10.1371/journal.pgph.0002068)
Supplement: S1 Checklist — (DOCX) [file pgph.0002068.s003.docx]

# STROBE Statement—checklist of items that should be included in reports of observational studies

|  | Item No. | Recommendation | Page  No. | Relevant text from manuscript |
| --- | --- | --- | --- | --- |
| **Title and abstract** | 1 | (*a*) Indicate the study’s design with a commonly used term in the title or the abstract | 1 | Syndromic surveillance during 2022 Uganda martyrs’ commemoration |
|  |  | (*b*) Provide in the abstract an informative and balanced summary of what was done and what was found | 1–2 | Mass gatherings frequently include close, prolonged interactions between people, which presents opportunities for infectious disease transmission. Over 20,000 pilgrims gathered at Namugongo Catholic and Protestant shrines to commemorate 2022 Uganda Martyr’s Day. We described syndromes suggestive of key priority diseases particularly COVID–19 and viral hemorrhagic fever (VHF) among visiting pilgrims during May 25−June 5, 2022. We conducted a survey among pilgrims at the catholic and protestant shrines based on signs and symptoms for key priority diseases: COVID–19 and VHF. A suspected COVID–19 case was defined as acute respiratory illness (temperature greater 37.5℃ and at least one sign/symptom of respiratory infection such as cough or shortness of breath) whereas a suspected VHF case was defined as fever >37.5^0^C and unexplained bleeding among pilgrims who visited Namugongo Catholic and Protestant shrines from May 25 to June 5, 2022. Pilgrims were sampled systematically at entrances and demarcated zonal areas to participate in the survey. Additionally, we extracted secondary data on pilgrims who sought emergency medical services from Health Management Information System registers. Descriptive analysis was conducted to identify syndromes suggestive of key priority diseases. Among 1,350 pilgrims interviewed, 767 (57%) were female. The mean age was 37.9 (±17.9) years. Nearly all pilgrims 1,331 (98.6%) were Ugandans. A total of 236 (18%) reported ≥1 case definition symptom and 42 (3%) reported ≥2 symptoms. Thirty-nine (2.9%) were suspected COVID–19 cases and three (0.2%) were suspected VHF cases from different regions of Uganda. Among 5,582 pilgrims who sought medical care from tents, 628 (11.3%) had suspected COVID–19 and one had suspected VHF. Almost one in fifty pilgrims at the 2022 Uganda Martyrs’ commemoration had at least one symptom of COVID–19 or VHF. Intensified syndromic surveillance and planned laboratory testing capacity at mass gatherings is important for early detection of public health emergencies that could stem from such events. |
| Introduction | | | |  |
| Background/rationale | 2 | Explain the scientific background and rationale for the investigation being reported | 2–4 | Occurrence of a disease outbreak at or during an international mass gathering has an increased potential for spreading to neighboring countries or even globally; thus, the need for reporting under the 2005 International Health Regulations. Influx of people during mass gatherings impacts a strain on existing surveillance and response systems. This presents a challenge to the hosting community or country to strengthen surveillance and response systems during preparation, operational and post-event phases of mass gatherings. Syndromic surveillance — the utilization of health-related data based on clinical observations and symptoms rather than confirmed diagnosis, can serve as an effective strategy for appropriate real time monitoring, early detection and timely response to public health events during mass gatherings. Every year, in June, pilgrims from Uganda and neighboring countries gather at Namugongo Catholic and Protestant shrines to commemorate the lives of Uganda Martyrs. Due to the distances people travel to attend this event, an infectious disease outbreak that starts during this mass gathering has high potential to spread to neighboring districts or even to other countries. In this context, we described syndromes suggestive of key priority diseases among visiting pilgrims from May 25 to June 5, 2022 to inform future planning for mass gatherings in Uganda. |
| Objectives | 3 | State specific objectives, including any prespecified hypotheses | 4 | We described syndromes suggestive of key priority diseases among visiting pilgrims from May 25 to June 5, 2022 to inform future planning for mass gatherings in Uganda. |
| Methods | | | |  |
| Study design | 4 | Present key elements of study design early in the paper | 4 | Cross-sectional study design |
| Setting | 5 | Describe the setting, locations, and relevant dates, including periods of recruitment, exposure, follow-up, and data collection | 4 | This assessment was conducted among over 20,000 visiting pilgrims from Uganda and neighboring countries gathered at Namugongo Catholic and Protestant shrines located in Namugongo Division, Wakiso District, Uganda. On–site emergency medical services were provided in designated tents from May 25 to June 5, 2022 by medical teams comprising emergency medicine specialists, doctors, nurses, laboratory attendants, and ambulance teams from Ministry of Health, Mulago National Referral Hospital, St. Francis Hospital Nsambya, Uganda Martyrs Hospital Rubaga, Uganda People’s Defence Forces, Uganda Police Force, Uganda Red Cross Society, St. John’s Ambulance, Holy Family Virika Hospital, Mengo Hospital, Zia Angellina Health Centre, and St. Stephens Hospital. |
| Participants | 6 | (*a*) *Cohort study*—Give the eligibility criteria, and the sources and methods of selection of participants. Describe methods of follow-up  ***Case-control study*—Give the eligibility criteria, and the sources and methods of case ascertainment and control selection. Give the rationale for the choice of cases and controls**  *Cross-sectional study*—Give the eligibility criteria, and the sources and methods of selection of participants | 7 | We utilized two different methods for data collection. First, we conducted a survey among pilgrims at the Catholic and Protestant shrines based on signs and symptoms for key priority diseases from June 2−5, 2022. We sampled systematically every 10th pilgrim in the line at main entrance gates. Other pilgrims were selected randomly from demarcated zonal areas. Overall, surveillance officers from Makerere University School of Public Health interviewed 1,350 pilgrims who voluntarily participated in the survey. Second, we conducted records review based on the on-site emergency medical services provided at the Catholic and Protestant shrines from May 25 to June 5, 2022. We extracted all the available data on 5,582 pilgrims who sought medical care from Health Management Information System registers for review including age, sex, district of residence, signs and symptoms or provisional diagnosis. |
|  |  | (*b*) *Cohort study*—For matched studies, give matching criteria and number of exposed and unexposed  *Case-control study*—For matched studies, give matching criteria and the number of controls per case | N/A |  |
| Variables | 7 | Clearly define all outcomes, exposures, predictors, potential confounders, and effect modifiers. Give diagnostic criteria, if applicable | 5 | At analysis phase, a suspected COVID–19 case was defined as acute respiratory illness (temperature greater 37.5℃ and at least one sign/symptom of respiratory infection such as cough or shortness of breath) whereas a suspected VHF case was defined as fever >37.5^0^C and unexplained bleeding among pilgrims who visited Namugongo Catholic and Protestant shrines from May 25 to June 5, 2022. |
| Data sources/ measurement | 8* | For each variable of interest, give sources of data and details of methods of assessment (measurement). Describe comparability of assessment methods if there is more than one group | 4–5 | We utilized two different methods for data collection. First, we conducted a survey among pilgrims at the Catholic and Protestant shrines based on signs and symptoms for key priority diseases from June 2−5, 2022. The data collection tool was developed in KoboCollect based on signs and symptoms for selected priority diseases: COVID–19 and VHFs inclusive of Ebola Virus Disease, Crimean Congo Hemorrhagic Fever, Yellow Fever, Rift Valley Fever, and Marburg Hemorrhagic Fever. Signs and symptoms investigated were based on suspect case definitions as per the National Technical Guidelines for Integrated Disease Surveillance and Response. Signs and symptoms under investigation were: fever (temperature >37.5^o^C), cough, flu, headache, generalized body weakness, difficulty in breathing, jaundice, fainting or sudden collapse, and unexplained bleeding. Any other signs and symptoms reported by the participants were also recorded by the surveillance officers.  We sampled systematically every 10th pilgrim in the line at main entrance gates. Other pilgrims were selected randomly from demarcated zonal areas. Verbal informed consent was obtained from participants prior to interviews. Overall, surveillance officers from Makerere University School of Public Health interviewed 1,350 pilgrims who voluntarily participated in the survey. Survey data were downloaded in the Excel (.xls) format from the KoboCollect server and processed for analysis. Second, we conducted records review based on the on-site emergency medical services provided at the Catholic and Protestant shrines from May 25 to June 5, 2022. We extracted all the available data on 5,582 pilgrims who sought medical care from Health Management Information System registers for review including age, sex, district of residence, signs and symptoms or provisional diagnosis. |
| Bias | 9 | Describe any efforts to address potential sources of bias | N/A |  |
| Study size | 10 | Explain how the study size was arrived at | N/A |  |

| Quantitative variables | 11 | Explain how quantitative variables were handled in the analyses. If applicable, describe which groupings were chosen and why | 5 | | At analysis phase, a suspected COVID–19 case was defined as acute respiratory illness (temperature greater 37.5℃ and at least one sign/symptom of respiratory infection such as cough or shortness of breath) whereas a suspected VHF case was defined as fever >37.5^0^C and unexplained bleeding among pilgrims who visited Namugongo Catholic and Protestant shrines from May 25 to June 5, 2022. |  |
| --- | --- | --- | --- | --- | --- | --- |
| Statistical methods | 12 | (a) Describe all statistical methods, including those used to control for confounding | | 5 | We conducted univariate data analysis using Epi Info 7 software (CDC, Atlanta, USA) to obtain frequencies of demographic characteristics and syndromes suggestive of public health emergencies among pilgrims who participated in the survey or sought care from the medical tents. Only syndromes suggestive of key priority diseases were of interest to the investigative team. At analysis phase, a suspected COVID–19 case was defined as acute respiratory illness (temperature greater 37.5℃ and at least one sign/symptom of respiratory infection such as cough or shortness of breath) whereas a suspected VHF case was defined as fever >37.5^0^C and unexplained bleeding among pilgrims who visited Namugongo Catholic and Protestant shrines from May 25 to June 5, 2022. | |
|  |  | (*b*) Describe any methods used to examine subgroups and interactions | N/A | |  |  |
|  |  | (*c*) Explain how missing data were addressed | N/A | |  |  |
|  |  | (*d*) *Cohort study*—If applicable, explain how loss to follow-up was addressed  *Case-control study*—If applicable, explain how matching of cases and controls was addressed  *Cross-sectional study*—If applicable, describe analytical methods taking account of sampling strategy | N/A | |  |  |
|  |  | (*e*) Describe any sensitivity analyses | N/A | |  |  |
| Results | | | | | | |
| Participants | 13* | (a) Report numbers of individuals at each stage of study—eg numbers potentially eligible, examined for eligibility, confirmed eligible, included in the study, completing follow-up, and analysed | 7 | | Tables 1 and 2 |  |
|  |  | (b) Give reasons for non-participation at each stage | N/A | |  |  |
|  |  | (c) Consider use of a flow diagram | N/A | |  |  |
| Descriptive data | 14* | (a) Give characteristics of study participants (eg demographic, clinical, social) and information on exposures and potential confounders | 7 | | Tables 1 and 2 |  |
|  |  | (b) Indicate number of participants with missing data for each variable of interest | N/A | |  |  |
|  |  | (c) *Cohort study*—Summarise follow-up time (eg, average and total amount) | N/A | |  |  |
| Outcome data | 15* | *Cohort study*—Report numbers of outcome events or summary measures over time | N/A | |  |  |
|  |  | *Case-control study—*Report numbers in each exposure category, or summary measures of exposure | N/A | |  |  |
|  |  | *Cross-sectional study—*Report numbers of outcome events or summary measures | 9–10 | | Figures 1 and 2 |  |
| Main results | 16 | (*a*) Give unadjusted estimates and, if applicable, confounder-adjusted estimates and their precision (eg, 95% confidence interval). Make clear which confounders were adjusted for and why they were included | 9–10 | | Among 1,350 pilgrims interviewed, 767 (57%) were female. The mean age was 37.9 (±17.9) years. Nearly all pilgrims 1,331 (98.6%) were Ugandans. A total of 236 (18%) reported ≥1 case definition symptom and 42 (3%) reported ≥2 symptoms. Thirty-nine (2.9%) were suspected COVID–19 cases and three (0.2%) were suspected VHF cases from different regions of Uganda. Among 5,582 pilgrims who sought medical care from tents, 628 (11.3%) had suspected COVID–19 and one had suspected VHF. |  |
|  |  | (*b*) Report category boundaries when continuous variables were categorized | N/A | |  |  |
|  |  | (*c*) If relevant, consider translating estimates of relative risk into absolute risk for a meaningful time period | N/A | |  |  |

Continued on next page

| Other analyses | 17 | Report other analyses done—eg analyses of subgroups and interactions, and sensitivity analyses | N/A |  |
| --- | --- | --- | --- | --- |
| Discussion | | | | |
| Key results | 18 | Summarise key results with reference to study objectives | 12 | Overall, we identified 4 Viral Hemorrhagic Fever and 560 COVID-19 suspected cases during the 2022 Uganda Martyrs’ commemoration. |
| Limitations | 19 | Discuss the limitations of the study, taking into account sources of potential bias or imprecision. Discuss both the direction and magnitude of any potential bias | 11 | There were only seventeen trained surveillance officers despite the masses at the Namugongo Protestant and Catholic shrines, who started administering the survey questionnaire on June 2, 2022 instead of having commenced on the May 25, 2022, at the time when pilgrims started gathering. Therefore, it was difficult to generalize the findings on the signs and symptoms for selected priority diseases to the entire population that converged during the 2022 Uganda Martyrs’ commemoration. Additionally, 1,786 out of 5,582 records did not have specified signs and symptoms but only had a provisional diagnosis based on the clinician’s assessment. We could not categorize these pilgrims under any of the key priority diseases since they did not have specified signs and symptoms; which could have underestimated the syndromes suggestive of key priority diseases. |
| Interpretation | 20 | Give a cautious overall interpretation of results considering objectives, limitations, multiplicity of analyses, results from similar studies, and other relevant evidence | 10–12 | Results were comprehensively interpreted in the discussion section |
| Generalisability | 21 | Discuss the generalisability (external validity) of the study results | N/A |  |
| Other information | |  | | |
| Funding | 22 | Give the source of funding and the role of the funders for the present study and, if applicable, for the original study on which the present article is based | 13 | This project was supported by President’s Emergency Plan for AIDS Relief (PEPFAR) through US Centers for Disease Control and Prevention Cooperative Agreement number GH001353 through Makerere University School of Public Health. Its contents are solely the responsibility of authors and do not necessarily represent the official views of US Centers for Disease Control and Prevention, Department of Health and Human Services, Makerere University School of Public Health, or Ministry of Health. The staff of the funding body provided technical guidance in design of the study, ethical clearance and collection, analysis, and interpretation of data, and writing the manuscript. |

*Give information separately for cases and controls in case-control studies and, if applicable, for exposed and unexposed groups in cohort and cross-sectional studies.

**Note:** An Explanation and Elaboration article discusses each checklist item and gives methodological background and published examples of transparent reporting. The STROBE checklist is best used in conjunction with this article (freely available on the Web sites of PLoS Medicine at http://www.plosmedicine.org/, Annals of Internal Medicine at http://www.annals.org/, and Epidemiology at http://www.epidem.com/). Information on the STROBE Initiative is available at www.strobe-statement.org.
