# Supplementary material for: Syndromic surveillance during 2022 Uganda Martyrs’ commemoration
Source: PLOS Glob Public Health. 2024 Jan 25;4(1):e0002068. doi: 10.1371/journal.pgph.0002068 (PMC10810525; doi:10.1371/journal.pgph.0002068)
Supplement: Attachment — Submitted filename: RESPONSE TO REVIEWERS _ SYNDROMIC SURVEILLANCE MANUSCRIPT.docx [file pgph.0002068.s004.docx]

**Syndromic surveillance during 2022 Uganda martyrs’ commemoration**

**Authors:** Mackline Ninsiima^1*^, Mercy W. Wanyana^1^, Thomas Kiggundu^1^, Patrick King^1^, Bernard Lubwama^2^, Richard Migisha^1^, Lilian Bulage^1^, Daniel Kadobera^1^, Alex Riolexus Ario^1^

**Institution affiliations**

^1^Uganda Public Health Fellowship Program, Uganda National Institute of Public Health, Kampala, Uganda

^2^Division of Integrated Epidemiology and Surveillance, Ministry of Health, Kampala, Uganda

**Correspondence*: Email:** [mninsiima@uniph.go.ug](mailto:mninsiima@uniph.go.ug), [nmackline@musph.ac.ug](mailto:nmackline@musph.ac.ug)

**Point-by-point response to reviewers**

| **Comment** | **Response** |
| --- | --- |
| **Journal requirements** | |
| - Please revise your statement in the 'Ethical considerations' section of your manuscript text | Thank you for raising this concern. We have revised this section as follows:  This investigation was in response to an annually commemorated mass gathering and was therefore determined to be non-research; accordingly (in reference to the memorandum of understanding, the activity was waived the process of the full Institutional Review Board). In our country, the Uganda National Institute of Public Health (UNIPH), falls under the Ministry of Health (MoH); in essence, the UNIPH is a subordinate authority to MoH. The MoH gave authority and directive to conduct syndromic surveillance during this religious event, then UNIPH approved the study and all the study protocols that were used. All methods were performed in accordance with the approval and administrative clearance without any ethical breach. |
| - In the ethics statement in the Methods, you have specified that verbal consent was obtained. Please provide additional details regarding how this consent was documented and witnessed, and state whether this was approved by the IRB | Verbal consent in English and the local language was sought from pilgrims before participation in the survey. Systematically sampled participants were informed about the rationale of the survey and that their participation was voluntary without any negative consequences in case they refused. In accordance to the study protocol approved by UNIPH, there was a question in the screening section of the survey questionnaire inquiring as to whether the systematically sampled participant verbally consented; and for those who did not, they were skipped to the next 10th pilgrim in the line at main entrance gates. |
| - Please ensure that funding Information and financial disclosure statement are matched. - In the Funding Information you indicated that no funding was received. Please revise the Funding Information field to reflect funding received. | We have clarified this section and indicated that the authors did not receive any specified funding for this project. |
| - Please provide separate figure files in .tif or .eps format only and remove any figures embedded in your manuscript file. | Noted with thanks. All figures have been removed from the manuscript and attached separately in .tif format. |
| - We have noticed that you have uploaded Supporting Information files, but you have not included a list of legends. Please add a full list of legends for your Supporting Information files after the references list | Based on your guidance, a list of legend of supporting information has been after the reference list as follows:   - Figure 1: Schema showing suspected priority diseases among pilgrims who participated in the survey during the Uganda Martyrs’ commemoration mass gathering, May 25–June 5, 2022 - Figure 2: Schema showing suspected priority diseases among pilgrims who sought medical care from medical tents during the Uganda Martyrs’ commemoration mass gathering, May 25–June 5, 2022 - Medical records - Survey data |
| - All PLOS journals now require all data underlying the findings described in their manuscript to be freely available to other researchers, either 1. In a public repository, 2. Within the manuscript itself, or 3. Uploaded as supplementary information | Thank you for this guidance. The data has been uploaded under supporting information |
| **Reviewer’s comments** | |
| - Does this manuscript meet PLOS Global Public Health’s [publication criteria](https://journals.plos.org/globalpublichealth/s/criteria-for-publication)? Is the manuscript technically sound, and do the data support the conclusions? The manuscript must describe methodologically and ethically rigorous research with conclusions that are appropriately drawn based on the data presented.   Reviewer #1: Yes | The authors are honestly grateful for the complement submitted by the reviewer. |
| - Has the statistical analysis been performed appropriately and rigorously?   Reviewer #1: No | Thank you for this complaint… This was largely a descriptive study whose rationale was to identify suspected priority diseases at a mass gathering and did not test any hypotheses. |
| - Have the authors made all data underlying the findings in their manuscript fully available (please refer to the Data Availability Statement at the start of the manuscript PDF file)?   Reviewer #1: No | Based on the journal requirements, the data has been uploaded under supporting information. |
| - Is the manuscript presented in an intelligible fashion and written in standard English? | The authors are honestly grateful for the complement submitted by the reviewer. Thank you… |
| **General**   - The manuscript presents a compelling analysis of syndromic surveillance at a large gathering event, which is an important contribution to the literature on mass gatherings and disease outbreaks. However, several areas of the manuscript require additional detail and clarification to enhance the scientific rigor and reproducibility of the research, as well as to improve the paper's contribution to the scholarly discourse. | With reference to your guidance, we believe that the revised version of this manuscript will enhance the scientific rigor and reproducibility of the research. |
| **Introduction**   - The authors should ensure that they fully acknowledge the contributions of seminal papers on syndromic surveillance during mass gatherings. References such as <https://doi.org/10.2196/publichealth.7313> and <https://doi.org/10.2196/16119> should be discussed and included in the paper. The authors should also consider including discussions on notable syndromic surveillance efforts such as the ones during Hajj. One suggested paper on this subject is <https://bmcinfectdis.biomedcentral.com/articles/10.1186/s12879-022-07559-0> | We have acknowledged the contribution of these seminal publications in our introduction section as follows:   - Participatory surveillance enhanced through utilization of digital technologies during mass gatherings has been demonstrated during the 2014 FIFA World Cup and 2016 Olympic Games - Following the COVID-19 pandemic, syndromic surveillance for respiratory tract infections was conducted among pilgrims who attended Hajj in 2021 |
| - The study's objective should also be clearly stated in the introduction. Readers should not have to infer the aim of the research. | The objective has been revised to:  “Therefore, we described syndromes suggestive of key priority diseases among visiting pilgrims from May 25 to June 5, 2022” |
| **Methods**   - The symptoms selected for surveillance could be reconsidered. The use of 'flu' as a symptom could be misleading, as syndromic surveillance typically employs broader categories such as Acute Respiratory Infections (ARIs). | With reference to Integrated Disease Surveillance and Response (IDSR) guidelines, a suspected COVID–19 case was defined as acute respiratory illness with fever >37.5^0^C, flu, cough, and difficulty in breathing. This indicated that flu was among the symptoms considered but not a stand-alone symptom to base on for suspected COVID-19 infection. |
| - The paper's handling of ethical considerations needs clarification. It is contradictory to state that the study did not involve human subjects while also describing the collection of verbal informed consent from participants. | Thank you for raising this concern. The ethical considerations section has been revised appropriately. |
| - The data analysis also refers to 'flu' as a symptom. Consistent with my earlier comment, I would recommend removing this term and instead categorizing under ARIs. | With reference to Integrated Disease Surveillance and Response (IDSR) guidelines, a suspected COVID–19 case was defined as acute respiratory illness with fever >37.5^0^C, flu, cough, and difficulty in breathing. This indicated that flu was among the symptoms considered but not a stand-alone symptom to base on for suspected COVID-19 infection. |
| - The age brackets used in tables 1 and 2 should be clearly explained in the methodology section. The parameters for grouping age can significantly impact the study's findings, and thus need to be transparent. | Thank you for raising this concern. Age categories were based on the ranking utilized by Uganda Bureau of Statistics. |
| - The paper could benefit from more advanced statistical analysis. The study, in its current form, is largely descriptive and fails to test any hypotheses. The limitations section should acknowledge this, and any other shortcomings of the study that were not previously mentioned. | In the limitation section, we have acknowledged the fact that we did not test any hypotheses. Thank you for your guidance. |
| - Overall, while the topic and approach are relevant and interesting, the manuscript requires significant clarification and augmentation to meet publication standards. Addressing these comments will make a stronger contribution to the discourse on syndromic surveillance during mass gatherings and potentially influence best practices during such events. | The authors are grateful to your comprehensive guidance. We have endeavored to address all the comments to meet publication standards. |
