# Supplementary material for: Syndromic surveillance during 2022 Uganda Martyrs’ commemoration
Source: PLOS Glob Public Health. 2024 Jan 25;4(1):e0002068. doi: 10.1371/journal.pgph.0002068 (PMC10810525; doi:10.1371/journal.pgph.0002068)
Supplement: Attachment — Submitted filename: RESPONSE TO REVIEWERS _ SYNDROMIC SURVEILLANCE MANUSCRIPT.docx [file pgph.0002068.s005.docx]

**Syndromic surveillance during 2022 Uganda martyrs’ commemoration**

**Authors:** Mackline Ninsiima^1*^, Mercy W. Wanyana^1^, Thomas Kiggundu^1^, Patrick King^1^, Bernard Lubwama^2^, Richard Migisha^1^, Lilian Bulage^1^, Daniel Kadobera^1^, Alex Riolexus Ario^1^

**Institution affiliations**

^1^Uganda Public Health Fellowship Program, Uganda National Institute of Public Health, Kampala, Uganda

^2^Division of Integrated Epidemiology and Surveillance, Ministry of Health, Kampala, Uganda

**Correspondence*: Email:** [mninsiima@uniph.go.ug](mailto:mninsiima@uniph.go.ug), [nmackline@musph.ac.ug](mailto:nmackline@musph.ac.ug)

**Point-by-point response to reviewers**

| **Comment** | **Response** |
| --- | --- |
| - Thank you for addressing the comments raised by the previous reviewer, which has improved the manuscript. However, a few more revisions are needed before this manuscript can be considered for publication. | Thank you for your feedback and acknowledgment of the revisions made to the manuscript. We will address the additional highlighted revisions to ensure that the manuscript meets the standards required for publication. |
| - The previous reviewer requested that you not list "flu" as a symptom of COVID-19, which is a concern that I also share, as influenza is not a symptom of COVID-19 -- did you mean to say "influenza-like illness?" "Acute respiratory illness" would also make sense instead of "flu," per Reviewer 1's suggestion.  Furthermore, per the case definition provided in Uganda's IDSR guidelines (see Pg 418), there is no mention of "flu" as a criterion for COVID-19: https://www.afro.who.int/sites/default/files/2021-09/2_Uganda%203rd%20IDSR%20Tech%20Guideline_PrintVersion_10Sep2021.pdf  As such, please review the analysis to ensure that the data presented are in line with the correct case definition. Please also cite the IDSR guidelines so readers can look up case definitions, or if a different case definition of COVID-19 was used, please provide a citation in the text. | Thank you for raising this concern. In accordance with the case definition outlined in the National Technical Guidelines for Integrated Disease Surveillance and Response, we have substituted the term "flu" with "acute respiratory illness" in our study. Following this modification, we have reanalyzed the data to align with the accurate case definition provided.  **Reference:** “<https://www.afro.who.int/sites/default/files/2021-09/2_Uganda%203rd%20IDSR%20Tech%20Guideline_PrintVersion_10Sep2021.pdf> (Page 418)”, |
| - The discussion and conclusion would benefit from some consideration of the implications of this analysis for public health policymaking and practice in Uganda. The conclusion acknowledges the importance of prioritizing intensified syndromic surveillance, but some elaboration would be beneficial. Consider adding a few sentences or a paragraph about how Uganda might improve public health planning for future mass gathering events (e.g. intra/after-action review of the 2022 Martyrs' Commemoration, simulation exercises, risk assessments) and/or engage with policymakers to ensure that more resources are allocated toward syndromic surveillance. Your thoughts on future studies/analyses on this topic would also be beneficial. | Thank you for providing insights to improve the manuscript. We have revised the discussion and conclusion by adding these statements:  Evidence generated from this assessment indicates potential health risks linked to mass gatherings. Uganda should conduct intra and after-action reviews of mass gatherings to identify strengths and weaknesses of existing surveillance and response systems, identify areas for improvement, and provide actionable recommendations. This could serve as a learning opportunity to enhance the country's readiness and response capabilities for future mass gatherings. Integration of simulation exercises will be essential to evaluate existing protocols, identify gaps and refine strategies for managing large-scale events. Conducting risk assessments prior to mass gatherings could guide the development of tailored strategies for mitigating health risks, including the allocation of resources for syndromic surveillance, healthcare infrastructure, and emergency response.  Based on the findings, policy makers should increase funding, conduct trainings, and construct infrastructure, enhancing the public health system's ability to monitor and respond to potential outbreaks during mass gatherings. Future studies should focus on validating syndromic surveillance methods for mass gatherings, exploring the effectiveness of specific interventions implemented in response to such events, and assessing the long-term health impacts on both attendees and the surrounding communities. Furthermore, there is need to investigate the socio-economic and cultural factors influencing the transmission of infectious diseases during mass gatherings, providing a more holistic understanding of the associated risks.  Ultimately, evidence generated from the 2022 Uganda Martyrs' Commemoration underscores the paramount importance of proactive public health planning and response strategies for mass gatherings. By diligently incorporating the valuable lessons learnt from this event, Uganda may not only enhance but also expand its capacity to protect the public during similar occasions in the future. |
| - In Line 177 of the revised manuscript, there is a reference to "Church X." I understand that the church may need to be kept anonymous, but "Church X" reads a bit awkwardly. I suggest stating "In 2020, a social gathering at a church in Omoro District, Northern Uganda provided an opportunity for a COVID-19..." instead. | Thanks for your guidance. We have rephrased the statement as follows:  “In 2020, a social gathering at a church in Omoro District, Northern Uganda provided an opportunity for a COVID–19 superspreading event.” |
